# ALPPS for Locally Advanced Intrahepatic Cholangiocarcinoma: Did Aggressive Surgery Lead to the Oncological Benefit? An International Multi-center Study

**DOI:** 10.1245/s10434-019-08192-z

**Published:** 2020-01-30

**Authors:** Jun Li, Mohamed Moustafa, Michael Linecker, Georg Lurje, Ivan Capobianco, Janine Baumgart, Francesca Ratti, Falk Rauchfuss, Deniz Balci, Eduardo Fernandes, Roberto Montalti, Ricardo Robles-Campos, Bergthor Bjornsson, Stefan A. Topp, Jiri Fronek, Chao Liu, Roger Wahba, Christiane Bruns, Stefan M. Brunner, Hans J. Schlitt, Asmus Heumann, Björn-Ole Stüben, Jakob R. Izbicki, Jan Bednarsch, Enrico Gringeri, Elisa Fasolo, Jens Rolinger, Jakub Kristek, Roberto Hernandez-Alejandro, Andreas Schnitzbauer, Natascha Nuessler, Michael R. Schön, Sergey Voskanyan, Athanasios S. Petrou, Oszkar Hahn, Yuji Soejima, Emilio Vicente, Carlos Castro-Benitez, René Adam, Federico Tomassini, Roberto Ivan Troisi, Alexandros Kantas, Karl Juergen Oldhafer, Victoria Ardiles, Eduardo de Santibanes, Massimo Malago, Pierre-Alain Clavien, Marco Vivarelli, Utz Settmacher, Luca Aldrighetti, Ulf Neumann, Henrik Petrowsky, Umberto Cillo, Hauke Lang, Silvio Nadalin

**Affiliations:** 1 Department of General, Visceral and Thoracic Surgery, University Medical Center Hamburg-Eppendorf, Hamburg, Germany; 2 Hepatobiliary Surgery and Liver Transplant Unit, University of Padua, Padua, Italy; 3 Swiss HPB and Transplantation Center, Department of Surgery and Transplantation, University Hospital Zurich, Zurich, Switzerland; 4 Department of Surgery and Transplantation, University Hospital RWTH Aachen, Aachen, Germany; 5 Department of General, Visceral and Transplantation Surgery, University Hospital Tuebingen, Tübingen, Germany; 6 Department of General, Visceral and Transplantation Surgery, University Hospital Mainz, Mainz, Germany; 7 Hepatobiliary Surgery Division, San Raffaele Hospital, Milan, Italy; 8 Department of General, Visceral and Vascular Surgery, University Hospital Jena, Jena, Germany; 9 Department of Surgery, Ankara University, Ankara, Turkey; 10 Department of Surgery, Federal University of Rio de Janeiro, Rio de Janeiro, Brazil; 11 Department of Surgery and Transplantation, São Lucas Hospital - Copacabana, Rio de Janeiro, Brazil; 12 Department of Public Health, Federico II University Hospital, Naples, Italy; 13 Department of Surgery, Virgen de la Arrixaca Hospital, IMIB-Arrixaca, Murcia, Spain; 14 Department of Surgery and Department of Clinical and Experimental Medicine, Linköping University, Linköping, Sweden; 15 Department of General, Visceral and Pediatric Surgery, University Hospital Düsseldorf, Düsseldorf, Germany; 16 Department of Transplant Surgery, Institute for Clinical and Experimental Medicine, Prague, Czech Republic; 17 Department of Anatomy, Second Faculty of Medicine, Charles University, Prague, Czech Republic; 18 Department of Hepato-Pancreato-Biliary Surgery, Sun Yat-Sen Memorial Hospital, Sun Yat-Sen University, Guangzhou, China; 19 Department of General, Visceral, Cancer and Transplantation Surgery, University Hospital of Cologne, Cologne, Germany; 20 Department of Surgery, University Medical Center Regensburg, Regensburg, Germany; 21 Division of Transplantation and Hepatobiliary Surgery, University of Rochester, Rochester, NY USA; 22 Department of General, Visceral and Transplantation Surgery, University Hospital Frankfurt, Frankfurt, Germany; 23 Department of General, Visceral and endocrine Surgery, München Klinik Neuperlach, Munich, Germany; 24 Klinikum Karlsruhe, Karlsruhe, Germany; 25 Center for Surgery and Transplantology, A.I. Burnazyan Russian State Scientific Center FMBC of FMBA, Moscow, Russia; 26 Department of General Surgery, Nicosia Teaching Hospital, Strovolos, Cyprus; 27 1st Department of Surgery, Semmelweis University, Budapest, Hungary; 28 Department of Surgery, Shinshu University School of Medicine, Matsumoto, Japan; 29 “Clara Campal” Oncological Center, Sanchinarro University Hospital, San Pablo University. CEU, Madrid, Spain; 30 Centre Hépato-Biliaire, AP-HP Hôpital Paul Brousse, Inserm U 935, Univ Paris-Saclay, Villejuif, France; 31 Department of Human Structure and Repair, Faculty of Medicine, Ghent University, Ghent, Belgium; 32 Department of Clinical Medicine and Surgery, Federico II University, Naples, Italy; 33 Department of Surgery, Division of HPB Surgery, Asklepios Hospital Barmbek, Semmelweis University Budapest, Campus Hamburg, Hamburg, Germany; 34 HPB Surgery and Liver Transplant Unit, Italian Hospital Buenos Aires, Buenos Aires, Argentina; 35 Department of Surgery, University College London, London, UK; 36 Hepatobiliary and Abdominal Transplantation Surgery, Department of Experimental and Clinical Medicine, Polytechnic University of Marche, Ancona, Italy

## Abstract

**Background:**

ALPPS is found to increase the resectability of primary and secondary liver malignancy at the advanced stage. The aim of the study was to verify the surgical and oncological outcome of ALPPS for intrahepatic cholangiocarcinoma (ICC).

**Methods:**

The study cohort was based on the ALPPS registry with patients from 31 international centers between August 2009 and January 2018. Propensity score matched patients receiving chemotherapy only were selected from the SEER database as controls for the survival analysis.

**Results:**

One hundred and two patients undergoing ALPPS were recruited, 99 completed the second stage with median inter-stage duration of 11 days. The median kinetic growth rate was 23 ml/day. R0 resection was achieved in 87 (85%). Initially high rates of morbidity and mortality decreased steadily to a 29% severe complication rate and 7% 90-day morbidity in the last 2 years. Post-hepatectomy liver failure remained the main cause of 90-day mortality. Multivariate analysis revealed insufficient future liver remnant at the stage-2 operation (FLR2) to be the only risk factor for severe complications (OR 2.91, *p* = 0.02). The propensity score matching analysis showed a superior overall survival in the ALPPS group compared to palliative chemotherapy (median overall survival: 26.4 months vs 14 months; 1-, 2-, and 3-year survival rates: 82.4%, 70.5% and 39.6% vs 51.2%, 21.4% and 11.3%, respectively, *p* < 0.01). The survival benefit, however, was not confirmed in the subgroup analysis for patients with insufficient FLR2 or multifocal ICC.

**Conclusion:**

ALPPS showed high efficacy in achieving R0 resections in locally advanced ICC. To get the most oncological benefit from this aggressive surgery, ALPPS would be restricted to patients with single lesions and sufficient FLR2.

Intrahepatic cholangiocarcinoma (ICC) is the second most common primary liver tumor and its incidence is increasing worldwide.^1^–^3^ Liver resection (LR), mostly major hepatectomy, is the gold standard treatment with curative intention.^4^–^6^ For patients presenting with unresectable ICC, systemic chemotherapy remains the mainstay palliative treatment modality without long-term survival.^7^,^8^ Five-year overall survival after liver resection has been reported in the range 22–44%,^9^ whilst approximately 58% present with tumor recurrence within 24 months.^10^ To judge the resectability, sufficient future liver remnant (FLR) is a main factor beside the possibility of vascular as well as biliary resections and reconstructions.^11^–^13^

In order to avoid post-hepatectomy liver failure (PHLF), various procedures, including two-stage hepatectomy and portal vein ligation known as associating liver partition and portal vein ligation for staged hepatectomy (ALPPS), have been performed to induce hypertrophy of the FLR. ALPPS was first reported by Schnitzbauer et al. in 2012.^14^,^15^ This innovative technique was rapidly adopted by hepatobiliary centers in the management of advanced liver tumors due to its promising high R0 resection rate.^16^–^19^ Nevertheless, some legitimate concerns were raised due to its high morbidity and mortality in comparison to conventional major hepatectomies.^20^–^23^ The mid- and long-term oncological outcomes of the ALPPS procedure performed for locally advanced ICC patients remain unverified. The potential oncological benefit of this aggressive surgical approach over alternative therapeutic modalities such as chemotherapy has not been investigated to date.

## Aim

The primary objective of this study was to investigate the oncological benefit of the ALPPS procedure for locally advanced ICC in comparison to chemotherapy. Knowing that ALPPS is associated with high morbidity and early mortality, identifying the risk factors as well as the subgroup of patients that might not benefit from this procedure was the secondary objective.

## Patients and Methods

### Study Design

The present cohort was composed of data derived from the International ALPPS Registry (ClinicalTrials.gov: NCT01924741). The study was approved by the Scientific Committee of the ALPPS Registry on June 18, 2017 (http://www.alpps.net/?q=node/88). Other hepatobiliary centers that were not on the registry at that time were encouraged to register themselves. Questionnaires were sent to all centers to complete the items required, especially perioperative outcome as well as long-term outcome of the ALPPS procedure for ICC patients. Since patients undergoing ALPPS were those usually regarded as unresectable and would have had palliative chemotherapy, a group of patients receiving only palliative chemotherapy in the Surveillance, Epidemiology, and End Results Program (SEER) database (https://seer.cancer.gov) was chosen as a comparison to study the oncological benefits of the ALPPS procedure.

The study consisted of three main parts: (1) analyzing the safety and efficacy (in terms of resectability) of ALPPS in a multi-centric database and identifying the risk factors for postoperative morbidity and 90-day mortality; (2) comparing the overall survival with a propensity-score matched group of patients who received palliative chemotherapy from a national database; and (3) a supplementary analysis was performed to identify a subgroup of patients who might not benefit from the ALPPS procedure.

### Study Population

The registry data of ALPPS was first exported for the latest analysis on November 11, 2017. All centers updated the information until May 22, 2019. Patients with primary ICC who received chemotherapy without surgical resection were retrieved from the SEER database.

### Variables Definition

For the ALPPS group, data on patient demographics, comorbidities, reason for performing ALPPS, volumetric data, procedure details, postoperative complications, liver parenchyma status, tumor pathology, and follow-up with details on recurrence and survival status were provided by the ALPPS registry as well as by the participating centers.

For the chemotherapy group, the SEER * Stat statistical software (version 8.3.2) was utilized to select the study population. In this version, data of patients classified by the 7th edition of the AJCC TNM staging system were available during the period from January 2010 till December 2013. The primary site code “C22.1” referred to the intrahepatic bile duct, and the ICDO-3 histology/behavior code “8160/3” was for cholangiocarcinoma. The chemotherapy data were labeled as “Yes” vs “No/unknown”. Only cases labeled “Yes” for chemotherapy were included in the study. The chemotherapy regimens were not described in detail. Patients with ICC were identified according to the International Classification of Diseases for Oncology, 3rd edition, ICD-O-3/WHO, 2008. The codes for patient demographics, tumor size, multifocality, vascular invasion, lymph node metastases and survival were interpreted using the Collaborative Stage data set (http://web2.facs.org/cstage0205/liver/Liverschema.html).

### Outcome Assessment

Volumetry study was represented by FLR to standard total liver volume^24^ (FLR/sTLV) ratio and FLR to body weight (FLR/BW) ratio. The median values of FLR/BW and FLR/sTLV after stage-1 and stage-2 hepatectomies were utilized as cut-off values for logistic regression analyses. To standardize kinetic growth, a mean volume (ml and %) increase per day was calculated assuming a linear growth model.^25^ Growth was expressed in FLR increase per day in percent. Complications were identified according to liver surgery specific clinical endpoints (CEP).^26^,^27^ The five elements of CEP beside mortality are PHLF, ascites, bile leak, infection and post hepatectomy hemorrhage (PHH). Complications were graded according to the definitions of the International Study Group of Liver Surgery (ISGLS) and the Clavien-Dindo classification.^28^–^31^ We defined severe complications as a Clavien-Dindo grade 3b or greater, including postoperative mortality (i.e., grade 5). The histological data consisted of TNM staging according to the 7th edition of AJCC staging system and tumor resection margin status. The follow-up data in the ALPPS group included the survival status, reason for death, recurrence status, time to recurrence, or death. Time to recurrence was defined as being from the stage-2 operation until recurrence (hepatic or extrahepatic). Overall survival (OS) was defined as the date of the first stage operation of ALPPS until death or last follow-up.

In the chemotherapy group the outcome included survival status and cause of death. The survival per month variable in the SEER data is explicitly reported in numbers, which represent the time from diagnosis to death (for deceased patients) or last follow-up (for alive patients). Cause of death was reported as “death due to cancer,” “death due to other cause,” or “unknown.”

### Statistical Analysis

Continuous variables are expressed as median (range) and were analyzed by the Mann–Whitney *U* test. Categorical-nominal variables are presented as a number (percentage) and were analyzed by the C2 or Fisher’s exact tests, as appropriate. All the represented percentages in the result section have excluded the missing values.

For the ALPPS group, uni- and multivariate logistic regression analyses were performed to verify risk factors for severe complications including the 90-day mortality. In order to understand the correlation between risk factors, the phi coefficient was calculated between nominal variables or between nominal variables and modified categorical variables. The Kaplan–Meier method was utilized to calculate overall survival and recurrence, and the log-rank test was used to assess the difference between curves. Cox proportional hazard regression was performed to evaluate risk factors associated with prognosis. Variables with *p* < 0.1 in the univariate analysis were further included in the multivariate Cox proportional hazards regression analysis with a stepwise forward conditional selection. Based on the propensity score, one-to-one nearest neighbor matching with replacement was adopted to overcome selection bias and minimize differences between the chemotherapy group and the ALPPS group. The propensity scores calculated by a logistic regression model represent the probability of each patient being assigned to each treatment. The variables included in this model were: age, gender, tumor stage, and lymph node status. Two-tailed *p* < 0.05 values were considered statistically significant and all statistical calculations were performed using SPSS version 21.0 (Chicago, IL, USA).

## Results

### Demographics and Clinical Features

A total of 102 patients with ICC undergoing ALPPS in 31 institutes from 18 countries between August 2009 and January 2018 were included. The median number of patients per center was two (range, 1–12). Ten centers performed ≥ 5 ALPPS for ICC each.

There were 46 men and 56 women, median age 65 years (32–84) (Table 1). The reason for performing ALPPS was insufficient FLR for one-stage hepatectomy according to the surgeon’s assessment. In 86.6% of patients the decision to perform the ALPPS procedure was taken solely according to the liver volume. Insufficient liver function due to diseased liver parenchyma with limited liver volume^32^ was mentioned in 5.2% of patients. No details were given in the other 8.2% of patients.

**Table 1:** Patients’ demographic and clinical features

Variable	Definition	All patients (*n* = 102)
Age		65 (32–84) years
	≤ 65 years	55 (53.9%)
	> 65 years	47 (46.1%)
Gender	Female	56 (54.9%)
	Male	46 (45.1%)
BMI		25.3 (16.3–38.3)
Diabetes mellitus	Yes	11 (11%)
Other comorbidities	Yes	26 (25.5%)
Preoperative chemotherapy	Yes	8 (7.9%)
Tumor location in preoperative imaging	1 = single lesion centrally located	70 (72.2%)
	2 = multiple tumors located in right liver lobe requiring extended right hepatectomy	18 (18.6%)
	3 = Bilobular tumors requiring clearance of FLR and right or extended right hepatectomy	9 (8.8%)
Surgical decision for ALPPS	1 = neither volume nor function of FLR sufficient	84 (86.6%)
	2 = volume sufficient but functional FLR insufficient	5 (5.2%)
	3 = not specified	8 (8.2%)
FLR stage 1 (FLR1)		336 (128–664) ml
FLR1/sTLV		22% (9–39%)
FLR1/BW		0.46% (0.19–0.84%)
FLR stage 2		611 (270–982) ml
FLR2/sTLV		40% (16–69%)
FLR2/BW		0.84% (0.35–1.51%)

*BMI* Body mass index, *BW* Body weight, *FLR* Future liver remnant, *sTLV* Standard total liver volume

Based on preoperative radiological assessment, a single lesion centrally located was reported in 72.2%. Multiple lesions in the right lobe were reported in 18.6%. Bilobular tumors requiring clearance of FLR was found in 8.8%. Preoperative chemotherapy with gemcitabine and cisplatin was carried out in 7.9% (Table 1). Preoperative biliary drainage by means of endoscopic retrograde cholangio-pancreatography (ERCP) or percutaneous transhepatic cholangiography (PTC) was performed in 5%.

### Procedure Details

For stage-1 operations intraoperative blood transfusion was reported in 22% of patients. Lymphadenectomy was performed in 67.4%. Tumor resection in the FLR was reported in 12.2%. Biliary digestive anastomosis was performed in 13.7%. Vascular reconstruction was reported in 2%. Full laparoscopic resection was performed in 6%. Hybrid ALPPS^33^ was performed in 5%. The median intensive care unit (ICU) stay after a stage-1 operation was 1 day (0–30).

In total, 99 patients underwent a stage-2 operation. The median inter-stage duration was 11 days (3–49). The stage-2 operation could not be carried out in three (2.9%) patients. Insufficient FLR hypertrophy as well as tumor progression were the reasons reported in two patients. The third patient died due to postoperative hemorrhage after a stage-1 operation.

In stage-2 operations intraoperative blood transfusion was reported in 33.1%. Right trisectionectomy was performed in 78.7% of cases. Right hepatectomy was performed in the remaining cases. Biliary digestive anastomosis was performed in 22%. Vascular reconstruction was performed in 12.1%. Full laparoscopic resection was performed in 9.6%. Lymphadenectomy was performed in 20.9%. The median ICU stay after a stage-2 operation was 1 day (0–43).

### Volumetry Study Outcome

For stage-1 operations the median FLR/sTLV ratio (FLR1/sTLV) was 22% (9–39%). The median FLR1/BW ratio was 0.46% (0.19–0.84%). For stage-2 operations the median FLR/sTLV ratio (FLR2/sTLV) was 40% (16–69%). The FLR increase was 75% (range, 3–192%). The median FLR2/BW ratio was 0.84% (0.35–1.51%). The median kinetic growth rate (KGR) was 7.3%/day (0.4–24.2%) or 23 ml/day (2–70). The median KGR was found to be higher in patients with age < 65 years compared to patients with age ≥ 65 years (8.7%/day vs 6.0%/day, *p* = 0.021). The quality of liver parenchyma was not confirmed as a factor influencing the KGR (*p* = 0.426).

### Postoperative Morbidity and Mortality (Table 2)

- After stage-1 operation:

**Table 2:** Liver surgery-specific complications after stage-1 and stage-2 operation

Complications	Definition	Stage-1 (*n* = 102)	Stage-2 (*n* = 99)
Liver failure	No	74/83 (89.2%)	56/86 (65.1%)
	PHLF grade A	6/83 (7.2%)	9/86 (10.5%)
	PHLF grade B	3/83 (3.6%)	10/86 (11.6%)
	PHLF grade C	0/83 (0%)	11/86 (12.8%)
Ascites	No or less than 500 ml/day after POD 3	60/72 (83.3%)	36/74 (48.7%)
	Grade A (over 500 ml/day)	8/72 (11.1%)	12/74 (16.2%)
	Grade B (requiring diuretics and/or albumin but less than 1000 ml/day after POD 7)	2/72 (2.8%)	11/74 (14.8%)
	Grade C (more than 1000 ml/day after POD 7)	2/72 (2.8%)	15/74 (20.3%)
Hemorrhage	No transfusion	70/95 (73.7%)	66/96 (68.7%)
	Grade A	8/95 (8.4%)	8/96 (8.3%)
	Grade B	2/95 (2.1%)	7/96 (7.3%)
	Grade C	3/95 (3.2%)	3/96 (3.1%)
	Reported as hemorrhage but without further detail	12/95 (12.6%)	12/96 (12.6%)
Infection complication	No	82/95 (86.3%)	54/96 (56.2%)
	Grade A (Clavien-Dindo grade II)	9/95 (9.5%)	12/96 (12.5%)
	Grade B (Clavien-Dindo grade IIIa)	3/95 (3.2%)	8/96 (8.3%)
	Grade C (Clavien-Dindo grade IIIb and more)	1/95 (1.0%)	12/96 (12.5%)
	Reported as infection but without further detail	0	10/96 (10.5%)
Bile leaks	No	83/95 (87.4%)	67/97 (69.1%)
	Grade A	9/95 (9.5%)	6/97 (6.2%)
	Grade B	3/95 (3.2%)	7/97 (7.2%)
	Grade C	0	7/97 (7.2%)
	Reported as bile leak but without further detail	0	10/97 (10.3%)

All the represented percentages in the Results Section have excluded the missing values

*PHLF* Post-hepatectomy liver failure

The overall morbidity after a stage-1 operation was 25%. Liver surgery specific complications were reported as PHLF in 10.8%, ascites in 16.7%, bile leak in 12.6%, infection complications in 13.7%, and PHH in 26.3%.

Severe complications (Clavien-Dindo grades 3b to 5) were reported in six patients (6.1%). Among them, three were infection complications and the other three were PHH. No grade C PHLF or bile leak according to the ISGLS classification were reported. One patient died on 13th day after the stage-1 operation due to PHH.

(b) After stage-2 operation:

The overall morbidity after a stage-2 operation was 76.8%. Liver surgery specific complications were reported as PHLF in 34.9%, ascites in 51.3%, bile leak in 30.9%, infection complications in 43.8%, and PHH in 31.3% (Table 2).

**Fig. 1:**
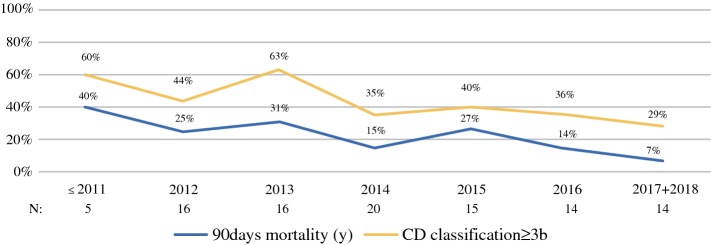
Severe postoperative complications (Clavien-Dindo classification 3b and 4) and 90-day mortality changes over years. The severe complication rate without 90-day mortality was 60% in the early years and has since dropped to 29% recently. The 90-day mortality decreased from 40% to 7%

Severe complications (Clavien-Dindo grades 3b to 5) were reported in 41.4% of the cases, including a 90-day mortality of 21.2% (21 patients). The morbidity and mortality rates steadily decreased over the years (Fig. 1). Among the severe complications, infection complication was reported in 12.5%. Grade C PHLF, bile leak, and PHH were reported in 12.8%, 7.2%, and 3.1%, respectively. Therapy refractory ascites (defined as a daily ascites volume more than 1000 ml despite medical treatment after POD 7) developed in 20.3% patients.

The causes of 90-day mortality were PHLF (*n* = 10), infection complication (*n* = 4), biliary complication (*n* = 2), early tumor recurrence (*n* = 4), and other surgical complication (duodenum perforation, *n* = 1).

### Risk Factors for Severe Complications (Clavien-Dindo Grades 3b to 5)

In the univariate analysis, statistically significant risk factors were age > 65 years, intraoperative blood transfusion in stage-1 operation and FLR2/BW ratio < 0.8%. In multivariate analysis a FLR2/BW ratio < 0.8% was the only statistically significant risk factor (OR 2.92, *p* = 0.02) for developing severe postoperative complications.

FLR2/sTLV < 40% or FLR2/BW ratio < 0.8% were found to be associated with PHLF by phi coefficient analysis with moderate negative relationships (phi = 0.307 and 0.375, respectively, *p* < 0.01).

Besides age > 65 years, FLR2/sTLV < 40%, FLR2/BW < 0.8% and elements of liver-surgery specific complications such as PHLF, ascites, infection, and PHH, but not bile leak, were associated with 90-day mortality by univariate logistic regression analysis. The multivariate analysis identified FLR2/sTLV < 40% as the only statistically significant risk factor for 90-day mortality (OR 5.52, *p* = 0.01).

### Histopathological Results

Among the studied population, adenocarcinoma was reported in 93.7% of cases (Table 3). Other subtypes of ICC were not specified. Positive lymph nodes were identified in 37.2% (*n* = 35). Tumor multifocality was reported in 60%. In non-tumor liver parenchyma, fibrosis was found in 26.2%, cirrhosis in 2.4%, and steatosis > 30% in 7.1% patients.

**Table 3:** Histopathological features of 99 patients with intrahepatic cholangiocarcinoma (ICC)

Variable	Definition	Patients (*n* = 99)
Histology	Adenocarcinoma	90/96 (93.7%)
	Other	6/96 (6.3%)
Margin	Negative of tumor	87/99 (87.9%)
	Positive of tumor	12/99 (12.1%)
Grading	1	7/87 (6.9%)
	2	52/87 (59.8%)
	3	29/87 (33.3%)
Stage (AJCC 7th edition)*	I	6/84 (7.1%)
	II	33/84 (39.3%)
	III	10/84 (11.9%)
	IVa	35/84 (41.7%)
Primary tumor*	T1	13/93 (14%)
	T2	38/93 (40.9%)
	T2a	6/93 (6.4%)
	T2b	14/93 (15%)
	T3	20/93 (21.5%)
	T4	2/93 (2.2%)
Nodal status*	N0	59/94 (62.8%)
	N1	35/94 (37.2%)
Metastasis*	M0	94/94 (100%)
	M1	0
Multifocal lesion	N	55/91 (60.4%)
	Y	36/91 (39.6%)
Number of lesions		1 (1–6)
Largest tumor size (mm)		85 (6–260)
Non-tumor Liver histology	Normal	51/84 (60.7%)
	Fibrosis	22/84 (26.2%)
	Steatosis > 30%	6/84 (7.1%)
	CASH	3/84 (3.6%)
	Cirrhosis	1/84 (2.4%)

All the represented percentages in the Results Section have excluded the missing values

*AJCC 7th edition of ICC was used for the TNM and tumor stage

*CASH* Chemotherapy associated steatotic hepatitis, *ICC* Intrahepatic cholangiocarcinoma

Based on the 7th edition of the AJCC staging system, the disease was classified as stage I in 7.1%, stage II in 39.3%, stage III in 11.9%, and stage IVa in 41.7%. A negative margin was achieved in 87 patients (87.9%).

### Follow-Up

The median follow-up by the reverse Kaplan–Meier method was 31.8 (17.4–46.2) months. Overall mortality was reported in 49.5% (*n* = 49). The median OS was 26.4 months. The overall survival rate at 1, 2, 3, and 5 years postoperatively was 64.3%, 52.5%, 38.8% and 22.0%, respectively. At the end of follow-up, tumor recurrence (including intra- and extrahepatic recurrence) was reported in 53 patients (55.8%). The median recurrence time was 9.3 months (5.9–12.73). The overall recurrence rate at 1, 2, and 3 years postoperatively was 55.1%, 74.3%, and 92%, respectively. The intrahepatic recurrence rate at 1, 2, and 3 years postoperatively was 49.7%, 68%, and 82.9%, respectively. The extrahepatic recurrence rate at 1, 2, and 3 years postoperatively was 29.1%, 48.2%, and 53.4%, respectively.

According to preoperative imaging, a subgroup analysis of two groups of patients was performed: Group A with single lesion (*n* = 70); Group B with multiple lesions (*n* = 27). The 1-, 2-, and 3-year recurrence rates were 54.2%, 71.5%, and 91.1% in Group A and 67.8%, 100%, and 100% in Group B, with a median recurrence time of 11 (6.6–15.5) months in Group A and 4.5 (3.1–6) months in Group B (*p* = 0.03). Group B had a higher rate of extrahepatic recurrence than Group A (1-, 2-, and 3-year recurrence rates were 51.1%, 77.7%, and 77.7% in Group B vs 22.6%, 39.4%, and 46.1% in Group A, *p* = 0.01) while the hepatic recurrence rate was similar in both groups.

### Propensity Score Matching Analysis

Data with TNM staging from the AJCC 7th edition were only available between January 2010 and December 2013 in the SEER database. Within this time period, 453 patients with pathological confirmation of ICC, who received chemotherapy only, were identified.

After propensity score matching by age, gender, tumor stage and lymph node status, 88 patients in each group were included for further analysis. The median age was 65 years in the ALPPS group and 64 years in the chemotherapy (CTx) group. The stage distribution of tumors was stage I in 10.2%, stage II in 38.6%, stage III in 13.6%, and stage IVa in 37.5% (Table 4).

**Table 4:** Propensity score matched patients of ALPPS group and chemotherapy (CTx) group

	Definition	CTx group (*n* = 88)	ALPPS group (*n* = 88)	*p* value
Age	Continuous	64 (36–85)	65 (32–81)	0.783
	≤ 65	45 (51.1%)	45 (51.1%)	1.000
	> 65	43 (48.9%)	43 (48.9%)	
Gender	Female	51 (58%)	51 (58%)	1.000
	Male	37 (42%)	37 (42%)	
Grade	G1	5 (8.8%)	7 (8.5%)	0.908
	G2	32 (56.4%)	49 (59.8%)	
	G3	21 (35.1%)	26 (31.7%)	
	N.A.	31 (35.2%)	4 (5%)	
Stage*	I	9 (10.2%)	9 (10.2%)	1.000
	II	34 (38.6%)	34 (38.6%)	
	III	12 (13.6%)	12 (13.6%)	
	IVa	33 (37.5%)	33 (37.5%)	
T*	T1	12 (13.6%)	12 (13.6%)	0.973
	T2	55 (62.5%)	55 (62.5%)	
	T3	18 (20.5%)	19 (21.6%)	
	T4	3 (3.4%)	2 (2.3%)	
N*	N0	57 (64.8%)	57 (64.8%)	1.000
	N1	31 (35.2%)	31 (35.2%)	
M*	M0	88 (100%)	88 (100%)	1.000
	M1	0–(0%)	0–(0%)	
Tumor Size (mm)	Continuous	75 (11–180)	85 (6–260)	0.204

*AJCC 7th edition was used for the TNM and tumor stage. All the represented percentages in the Results Section have excluded the missing values

*ALPPS* Associating liver partition and portal vein ligation for staged hepatectomy, *CTx* Chemotherapy

**Fig. 2:**
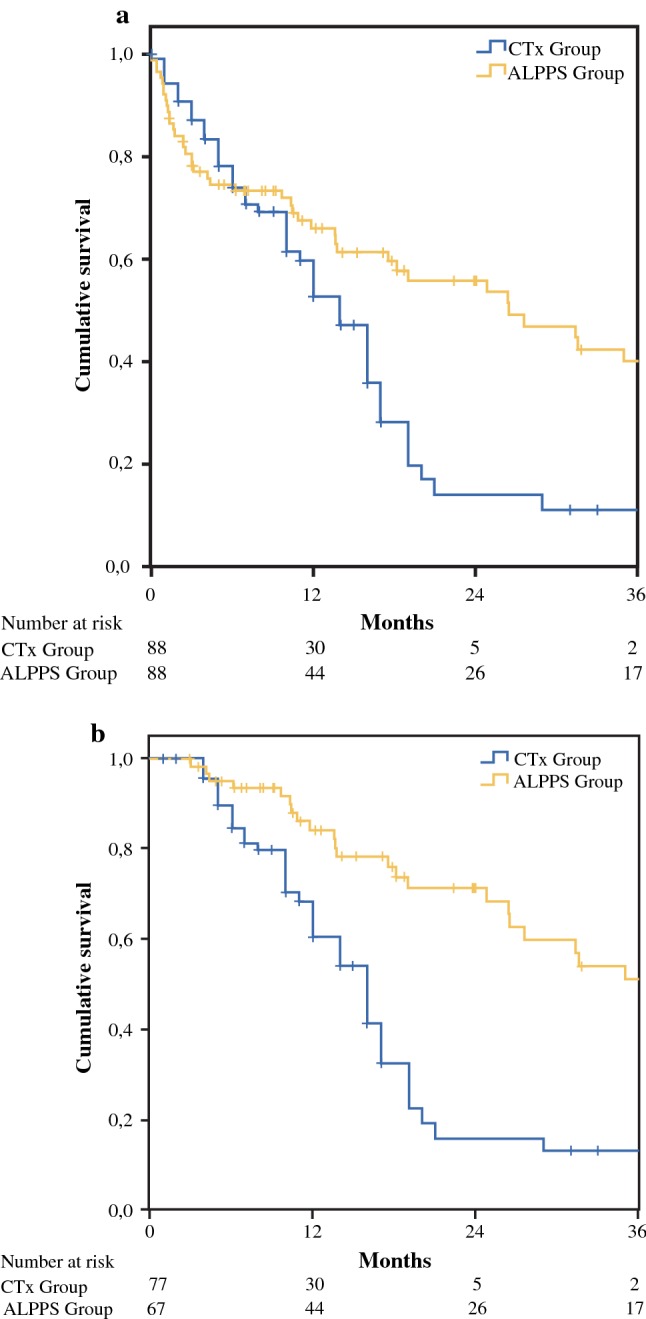
Overall survival in ALPPS group and chemotherapy (CTx) group. A superior overall survival was found in the ALPPS group despite higher 90-day mortality (*p* < 0.01). The difference in 2-year overall survival was 41.8% between the CTx group and the ALPPS group (**a**). The difference was 55.2% when 90-day mortality was excluded (**b**)

The 90-day mortality in the ALPPS group was higher than in the CTx group (21.8% vs 12.9%). Despite this, a superior OS was found in the ALPPS group with a median OS of 26.4 (12.6–40.2) months compared to 14 (11.4–16.6) months in the CTx group. The 1-, 2-, and 3-year survival rates were 66.0%, 55.9%, and 40.1% in the ALPPS group and 52.6%, 14.1%, and 11.3% in the CTx group, respectively (*p* < 0.01, Fig. 2).

By multivariate COX regression survival analyses, three statistically significant prognostic factors for poor overall survival were identified: (1) patients receiving chemotherapy only (HR = 1.734, *p* = 0.012); (2) age > 65 years (HR 1.561, *p* = 0.034); and (3) lymph node metastasis (HR 1.653, *p* = 0.022).

**Fig. 3:**
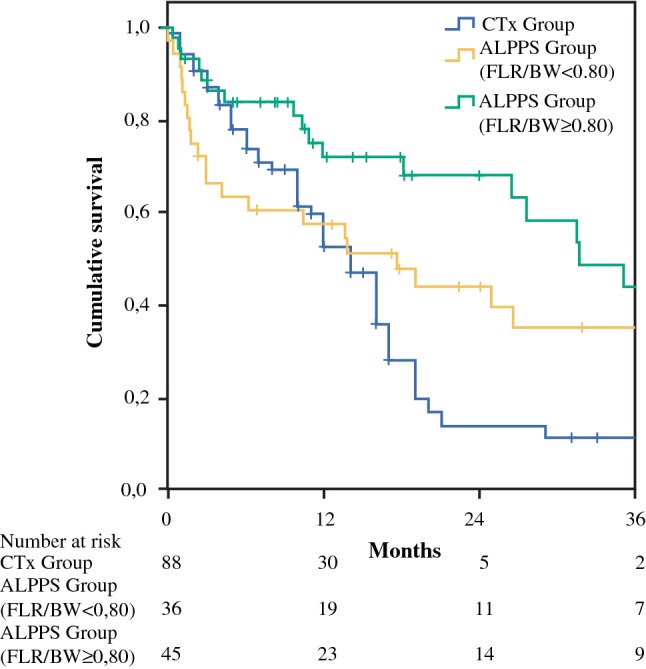
Overall survival analysis according to FLR2/BW with 0.8% as the cutoff. The benefit of ALPPS on overall survival was confirmed in the subgroup with FLR2/BW ratio ≥ 0.8% (*p* < 0.001) but not in FLR2/BW < 0.8% (*p* = 0.231) compared to the CTx group

As a FLR2/BW ratio less than 0.8% was the only significant risk factor for Clavien-Dindo grade ≥ 3b complication in the multivariate analysis (OR 2.91, *p* = 0.02), a subgroup analysis with FLR2/BW as the cut-off was analyzed. The benefit of ALPPS in regard to OS was confirmed in the subgroup with FLR2/BW ratio ≥ 0.8% (*p* < 0.001) but not in the subgroup of FLR2/BW < 0.8% (*p* = 0.231) compared to the CTx group (Fig. 3).

**Fig. 4:**
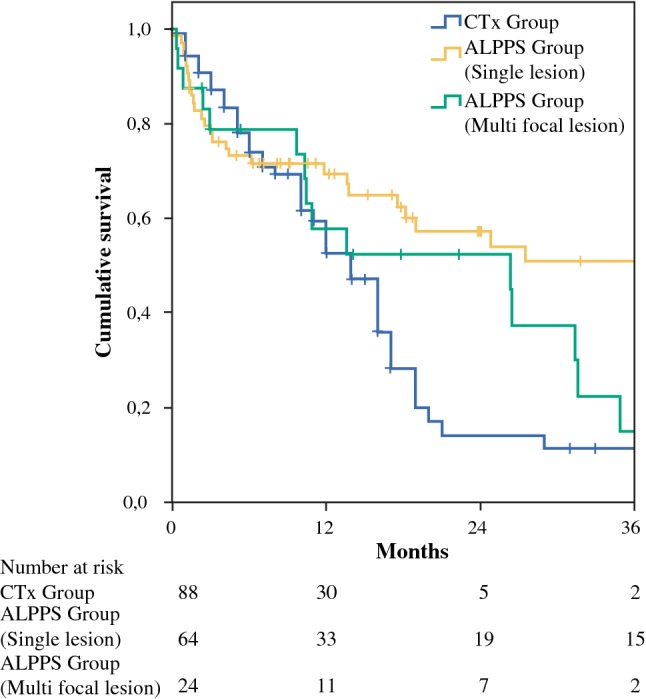
Overall survival analysis according to the number of lesions. The survival benefit of ALPPS over chemotherapy only can be found in patients with a single lesion (group A, *p* = 0.004) but not in patients with multiple lesions (group B, *p* = 0.247)

When the study population was divided according to preoperative imaging as Group A with a single lesion with the tumor centrally located and Group B with multiple lesions, the benefit of ALPPS on OS was confirmed in Group A (*p* = 0.004) but not in Group B (*p* = 0.247) when compared to the CTx group (Fig. 4).

## Discussion

Recently, ALPPS has been introduced as a novel solution that enables curative resection in marginally resectable tumors.^34^ Reports of the short-term outcomes of ALPPS in colorectal liver metastasis (CRLM), hepatocellular carcinoma (HCC), perihilar cholangiocarcinoma (CCA), but not ICC, have been published in the past.^20^,^23^,^35^,^36^ A look into the current literature reveals that definitive evidence showing a benefit of the ALPPS procedure in terms of survival and oncological outcome in patients with ICC is still lacking. Additionally, there is no large comparative study available to evaluate the risk–benefit balance of the ALPPS procedure in comparison to standard chemotherapy.

The results of our comparative survival analysis provide evidence for superior long-term outcomes of the ALPPS procedure over chemotherapy only. Despite the 90-day mortality, ALPPS shows superior 3-year overall survival rates compared to chemotherapy only (39.6% vs 11.3%, *p* = 0.01). The benefit of overall survival at 2 years was even greater, with a difference of 41.8% between the two groups (Fig. 2). Therefore, we believe that extensive liver resection by ALPPS procedure could provide better outcomes for patients with locally advanced ICC lesions, who are usually directed toward palliative chemotherapy. Long-term survival in this group of patients has also been achieved: the first ALPPS patient at the University Hospital Mainz celebrated her 10-year survival in August 2019. Except for an early intrahepatic recurrence during the 1st postoperative year, which was treated by repeated liver resection, the patient is now still disease free.

High morbidity and mortality rates after stage-2 operations were observed in patients with ICC, comparable with morbidity and mortality rates following ALPPS for other primary liver malignancies, such as HCC or perihilar CCA.^20^,^23^ The high complexity of surgery in this group of patients may be one of the main reasons for this observation. Indeed, trisectionectomy was performed in 78.7% of cases, biliary reconstruction in 35.6% and complex vascular reconstruction in 12.1%. Liver surgery specific complications were reported as PHLF in 30.3%, ascites in 49.3%, bile leak in 30.3%, infection complications in 42%, and PHH in 35.3%. Interestingly, when analyzing the risk factors for developing severe complications, lower future liver remnant volume at the stage-2 operation (FLR2), represented either by FLR2/BW < 0.8% or FLR2/sTLV < 40%, was the only statistically significant factor in the multivariate analysis. Moreover, FLR2/BW < 0.8% or FLR2/sTLV < 40%, was found to be correlated with PHLF (*p* < 0.01). Till now there has been no recommendation for defining the threshold FLR volume heading into a stage-2 operation in ALPPS. A FLR/BW ≥ 0.5% in a non-cirrhotic liver is usually deemed as safe in one-stage hepatectomy.^37^ The cut-off of FLR/BW for a safe liver resection was indicated to be 0.8% in this study, much more liver volume than 0.5% in one-stage hepatectomy. It further supported the conclusion that liver volume does not necessarily equal liver function in the setting of ALPPS.^38^ Recently, 99mTc-mebrofenin hepatobiliary scintigraphy has been reported to be a valuable technique to estimate the risk of PHLF.^39^,^40^ In ALPPS, a liver function test has been applied in several institutes to ensure a safe outcome.^41^ However, a clear cut-off is still not available. Moreover, the liver function test by 99mTc-mebrofenin hepatobiliary scintigraphy was not routinely performed at the majority of centers at the time of the current study. Based on the data on liver volume, the current study demonstrated that FLR2/BW < 0.8% or FLR2/sTLV < 40% was a risk factor for PHLF and severe complications. Thus, a threshold of FLR2/BW as 0.8% or FLR2/sTLV as 40% is recommended in ICC patients waiting for stage-2 operation whenever liver function tests are not available.

The 90-day mortality of 20.8% in our study is undisputedly high in comparison with mortality rates for conventional major hepatectomies or ALPPS performed for non-primary hepatobiliary malignancies.^18^,^21^,^42^ We would like to emphasize that the reported results of early mortality in our cohort represent both initial and recent experiences. In 2017 and 2018, the 90-day mortality was 7% (1/14), which had dropped significantly from 40% before 2012. We therefore conclude that the learning curve and improved patient selection will eventually lead to acceptable mortality rates.

The high rate of severe postoperative complications also had a significant impact on the survival benefit of the ALPPS procedure over chemotherapy only. Among the propensity score-match (PSM) analysis, the 90-day mortality in the ALPPS group was higher than in the CTx group (21.6% vs 12.5%). Despite this, a superior overall survival was found in the ALPPS group with a median survival time of 26.4 months compared to 14 months in the CTx group. Therefore, minimizing severe postoperative complications, especially the 90-day mortality, was among the most important tasks of ALPPS development. During recent years, major focus has been put on the surgical refinement of stage1 operations.^42^ A variety of methods for liver partition have been proposed.^17^,^43^–^45^ Accordingly, postoperative morbidity and mortality rates have been reduced.^42^ However, the importance of sufficient FLR at stage-2 has not been previously emphasized. In the current study, we found that a small FLR at stage-2 was the only statistically significant risk factor regarding 90-day mortality. In PSM analysis, the benefit of ALPPS on overall survival was confirmed in the subgroup with FLR2/BW ratio ≥ 0.8% (*p* = 0.001) but not in the subgroup of FLR2/BW < 0.8% (*p* = 0.294) in comparison to the chemotherapy only group. Indeed, achieving complete tumor resection (R0 in 87.9%) in this study arguably led to higher postoperative morbidity and mortality rates. Decision making for the timing of stage-2 operations remains a major challenge, especially when quantitative liver function tests are not available. Caution should be taken when the FLR/BW ratio prior to stage-2 operation is below 0.8%. This may well be the most important lesson learned over the course of this study.

The risk for high postoperative morbidity and mortality rates in the ALPPS procedure with R0 resection should be weighed against alternative approaches. Until now, portal vein embolization (PVE) has been the mainstay for inducing FLR hypertrophy preoperatively in many institutions, whereas the superiority of ALPPS to PVE has been demonstrated in patients with CRLM.^36^ In the majority of cases in this cohort, PVE would have been technically very demanding or even impossible due to the tumor mass and location. A propensity score-match to compare ALPPS with PVE in this group of patients was unfortunately not feasible due to the small numbers in both groups. However, the choice of either ALPPS or PVE in the treatment of ICC needs to be investigated. Furthermore, the role of neoadjuvent chemotherapy with or without PVE in selected patients should be studied. Locoregional treatment strategies for advanced ICC include transarterial chemoembolization, yttrium-labeled selective internal radiation therapy, and hepatic arterial infusion.^46^,^47^ Despite promising results reported, the conclusive evidence for efficacy of these strategies is still lacking.^1^,^8^

In a large multi-institutional series of 301 patients with ICC, more than half of the patients experienced recurrence after resection.^48^ The majority of patients in that cohort had T1 category tumors (58.1%). In the current cohort the patients suffered from more advanced tumor disease, with 53.4% showing AJCC stage III or IVa (T1 tumors were found only in 14%; 60.2% patients had multiple lesions; lymph nodes metastases were found in 40.7%). The advanced tumor stage led to a high tumor recurrence rate of 65.6% with a median recurrence time of 8 months. Intrahepatic recurrence was observed more frequently than extrahepatic recurrence. Patients with multiple lesions developed intrahepatic tumor recurrence earlier (4.5 vs 9 months in median) as well as more extrahepatic recurrence when compared to patients with a single lesion (52.2% vs 23%). In a recent meta-analysis of 4756 patients, ICC with multiple lesions was confirmed as one of the most important factors predicting shorter overall survival.^6^ In the current study with PSM analysis, the benefit of ALPPS on overall survival was only confirmed in patients with single lesion, not in patients with multiple lesions, in comparison to systemic chemotherapy. Therefore, because of high postoperative morbidity and mortality rates as well as the limited benefit of adjuvant chemotherapy,^8^ extensive liver resection using ALPPS could not be recommended as a better option than chemotherapy for ICC patients with multiple lesions. Since multiple lesions could represent systemic hematogenous metastatic intrahepatic dissemination or true multifocal disease, the prognostic relevance of them might be different. The former is definitely not an appropriate resection candidate.

The main limitation of this study is the retrospective methodology that leads to selection bias in both groups, especially that caused by under-reported cases with poor outcomes in patients undergoing ALPPS. The difference in experience of total cases of ALPPS per center as well as number of ICC patients might also have an influence on outcomes. Nevertheless, the use of propensity score matched analysis along with multivariate COX proportional hazard modeling has strengthened the degree of evidence and reduces this bias, and is more robustly precise than standard multivariable methods. The classifications used for the propensity score matching are pT and pN for the ALPPS group vs cT and cN for the chemotherapy-only group. This may underestimate the tumor stage in chemotherapy-only patients and lead to a negative selection bias. Moreover, comorbidity as well as Eastern Cooperative Oncology Group (ECOG) status was not included in the propensity score matching, which may lead to an overestimation of the overall survival in the ALPPS group. A randomized control trial to investigate short- and long-term outcomes would provide more reliable results.

We have utilized the AJCC staging system, 7th edition, in our analysis that was in line with the staging system utilized in both the ALPPS and SEER databases. Recently, the AJCC has released a new staging system for ICC in its 8th edition. Nevertheless, the 8th edition staging system for ICC has not shown a significant advantage over the 7th edition in overall prognostic discrimination except for stage III, which represent only 8.7% of patients included in this study.^49^

From another aspect, a major advantage of this study was utilizing the ALPPS registry database as a baseline to create an international ALPPS prospective cohort with a longitudinal study design by retrieving new data on the ALPPS approach not reported by the registry web site. This advantage will facilitate the establishment of further studies to investigate long-term oncological outcomes of the ALPPS procedure and allow for risk adjustment analyses. Further work is needed to achieve significant improvements in regard to the quality of the data available for patients undergoing ALPPS. Therefore, we urge all surgeons performing this procedure to share their experiences and data through registration in the ALPPS registry (www.alpps.net).

In conclusion, this multicentric study demonstrated a high resectability of 97% and R0 resection of 85.3% in 102 patients with initially “unresectable” locally advanced ICC by ALPPS. Superior 1-, 2-, and 3-year overall survival rates were found in the ALPPS group compared with the chemotherapy only group, despite high 90-day mortality rates. Better patient selection and improved interstage decision making over time contributed to the decrease in mortality with time. PHLF was observed in 35% of the patients, with 12.8% classified as grade C. A threshold of FLR/BW of 0.8% (or 40% if FLR/sTLV is used) is recommended in patients waiting for the stage-2 operation whenever liver function tests are not available. The benefit of ALPPS on overall survival was confirmed in ICC patients with a centrally located single lesion, but not for patients with multiple lesions when compared to chemotherapy only.
